# Effect of the XRCC1 codon 399 polymorphism on the repair of vinyl chloride metabolite-induced DNA damage

**DOI:** 10.4103/1477-3163.56290

**Published:** 2009-10-07

**Authors:** Yongliang Li, Changmin Long, George Lin, Marie-Jeanne Marion, Greg Freyer, Regina M. Santella, Paul W. Brandt-Rauf

**Affiliations:** 1 Department of Environmental and Occupational Health Sciences, School of Public Health, University of Illinois at Chicago, Chicago, IL 60612, USA; 2 Department of Unite de Recherche Virus des Hepatites et Pathologies Associee, INSERM, Lyon, France; 3 Department of Environmental Health Sciences, Mailman School of Public Health, Columbia University, New York, NY 10032, USA

**Keywords:** Base excision repair, chloroethylene oxide, etheno adducts

## Abstract

**Background:**

Recent epidemiologic evidence suggests that the common polymorphism at amino acid residue 399 of the x-ray cross complementing-1 (XRCC1) protein, a key component of the base excision repair (BER) pathway for DNA damage, plays a significant role in the genetic variability of individuals in terms of the mutagenic damage they experience following exposure to the carcinogen vinyl chloride (VC). The aim of this study was to provide support for the biological plausibility of these epidemiologic observations with experimental data derived from cell lines in culture from individuals who were either homozygous wild-type or homozygous variant for this XRCC1 polymorphism following exposure to chloroethylene oxide (CEO), the active metabolite of VC, with measurement of the induced etheno-DNA adducts before and after repair.

**Materials and Methods:**

Immortalized lymphoblast cell lines from seven VC workers (four homozygous wild-type and three homozygous variant for the 399 XRCC1 polymorphism) were exposed to CEO, and etheno-adenosine (εA) adduct levels were determined by enzyme-linked immunosorbent assay (ELISA) pre-exposure and at 0, 4, 8 and 24 h following exposure.

**Results:**

The average εA adduct levels were statistically significantly higher in the variant cells compared to the wild-type cells at 8 and 24 h following exposure (*P*< 0.05) with an overall average repair efficiency of 32% in the variant cells compared to 82% in the wild-type cells.

**Conclusion:**

These results are consistent with the epidemiologic findings of the types of VC-induced biomarkers observed in exposed individuals and the mutational spectra found in the resultant tumors as well as the key role that BER, especially XRCC1, plays in this carcinogenic pathway.

## INTRODUCTION

Vinyl chloride (VC) is a known animal and human carcinogen associated with the sentinel neoplasm of angiosarcoma of the liver (ASL). VC is used in large quantities around the world, primarily in the manufacture of polyvinyl chloride polymers. VC exposure occurs in the workplace environment during this manufacture and in the general environment from releases near plastics industries, hazardous waste sites and landfills.[1]

Following exposure, VC is metabolized in the liver to the reactive intermediates chloroethylene oxide (CEO) and chloracetaldehyde (CAA) that can interact with DNA and generate pro-mutagenic DNA adducts including etheno-guanosine (εG) and etheno-adenosine (εA).[1] Although these etheno-DNA adducts are capable of causing several different types of DNA mutations, they are known to be able to produce the particular types of K-*ras* oncogene mutations (G to A transitions) and *TP53* tumor suppressor gene mutations (A to T transversions) found in the ASLs of exposed workers.[2,3] We have previously shown that these mutations lead to the production of mutant oncoprotein biomarkers (mutant *ras*-p21 and mutant p53) that can be identified in the serum of VC-exposed individuals with ASLs as well as VC-exposed individuals without any detectable neoplastic disease in a highly statistically significant dose-response relationship with regard to estimated, cumulative VC exposure.[4,5] However, at any given VC exposure level there were individuals who were negative for both biomarkers, positive for one or the other biomarker, or positive for both biomarkers, suggesting that there might be some genetically determined susceptibility to VC mutagenesis that could account for different mutant biomarker outcomes with similar exposures. Recently, we have found that a polymorphism at amino acid residue 399 in the x-ray cross complementing- 1 (XRCC1) protein is a potential contributor to this variable susceptibility, especially in terms of the effect on the mutant p53 biomarker presumed to be caused by the εA adducts.[6,7] This is consistent with the fact that the types of pro-mutagenic adducts generated by VC exposure (especially εA) would be expected to be removed by base excision repair (BER), a process in which XRCC1 plays a key role.[8]

The purpose of the present study was to provide support for the biological plausibility of these *in vivo* molecular epidemiologic observations. This was to be accomplished through controlled *in vitro* experiments in which immortalized lymphoblast cell lines from the VC workers with different *XRCC1* genotypes were exposed to the active metabolite of VC (CEO) and the differences in efficiency of BER determined by measuring the effects on etheno-DNA adduct levels.

## MATERIALS AND METHODS

From the previously described population of VC-exposed workers in France who had been genotyped for the *XRCC1* 399 polymorphism,[4] immortalized lymphoblast cell lines were established by routine transformation techniques using an Epstein-Barr virus for four workers who were homozygous wild-type and three workers who were homozygous variant for this polymorphism. These two different subgroups of workers were otherwise similar to each other in terms of potentially confounding factors that could influence VC metabolism or repair of VC-induced damage, including age (all middle-aged), gender (all male), ethnicity (all Caucasian), alcohol consumption (mostly non-drinkers), or other relevant genotypes such as for *CYP2E1*, *ALDH2*, *XPD* and other polymorphic sites of *XRCC1* (all wild-type). This research was approved by the UIC Institutional Review Board.

For exposure of each cell line, 20 × 10^6^ cells were suspended in 20 mL of RPMI 1640 medium and incubated with CEO, the reactive intermediate of VC, at a concentration of 25 μg/mL for 1 h at 37° C (determined to be the optimum conditions for adduct generation with minimal cell lethality).[6] The cells were then washed with PBS and allowed to recover for 4, 8 or 24 h in RPMI 1640 containing 100 U/mL penicillin, 100 μg/mL streptomycin, 2 mM L-glutamine and 10% heat-inactivated fetal bovine serum to allow for DNA repair. For each time point (pre-exposure baseline, 0, 4, 8 and 24 h post-exposure), the cells were spun down and re-suspended in 1.5 mL TE buffer, pH 7.9, with 100 μL of 10% SDS, 20 μL of 10 mg/mL RNase A and 30 μL of 1000 U/mL of RNase T1, and incubated for 40 min at 37° C. Then 30 μL of 10 mg/mL proteinase K was added, followed by incubation for 1 h at 60° C and 40 min at 37° C and subsequent phenol/chloroform DNA extraction by routine techniques.[9]

The level of εA DNA adducts in equal amounts of DNA extracts (50 μg) from each cell line at each phase of treatment was quantitated using an εA-specific enzyme-linked immunosorbent assay (ELISA), as modified from the previously described protocol.[10] In this case, microtiter wells were coated with 0.4 ng of CAA-treated DNA, dried for 7 h at 37° C, washed, blocked, and then incubated with the test mixture (50 μL of 1:2000 1G4 primary antibody plus 50 μL of 1 μg/μL DNA from the treated cells or 50 μL of εdA standard (Sigma, St. Louis, MO) in 1 μg/μL CT DNA) for 1.5 h at 37° C. After washing, 100 μL of 1:600 secondary antibody solution (Biotin-SP-conjugated Goat Anti-mouse IgG_1_; Jackson ImmunoResearch Laboratories, West Grove, PA) was added to each well and incubated for 1.5 h at 37° C followed by washing and incubation with 100 μL of 1:10,000 conjugate solution (Aridx-APstreptavidin-Alkaline Phosphatase; Applied Biosystems, Foster City, CA) for 1 h at room temperature. After washing again, each well was incubated with 100 μL of substrate solution (CDP-Star with Emerald II; Applied Biosystems, Foster City, CA) for 30 min at room temperature followed by analysis on a microplate reader (Victor 2; Perkin Elmer, Waltham, MA). This ELISA is based on a mouse monoclonal antibody (1G4) raised against εA coupled to a carrier and shows no cross-reactivity with unmodified DNA, normal nucleotides or other etheno-DNA adducts. Adduct levels for each of the cell samples (run in duplicate) were derived by interpolation from a standard curve generated with known amounts of adducts. For statistical comparisons, the average adduct levels (in ng adduct/50 μg DNA) between the subgroups of workers (homozygous wild-type vs. homozygous variant) were compared at each time point using the t test. In addition, the average overall efficiency of DNA repair for the two groups of cell lines was calculated from the ratio of adducts removed during the recovery period to the amount of adducts formed from the exposure, corrected for the baseline levels.

## RESULTS

**Figure 1:**
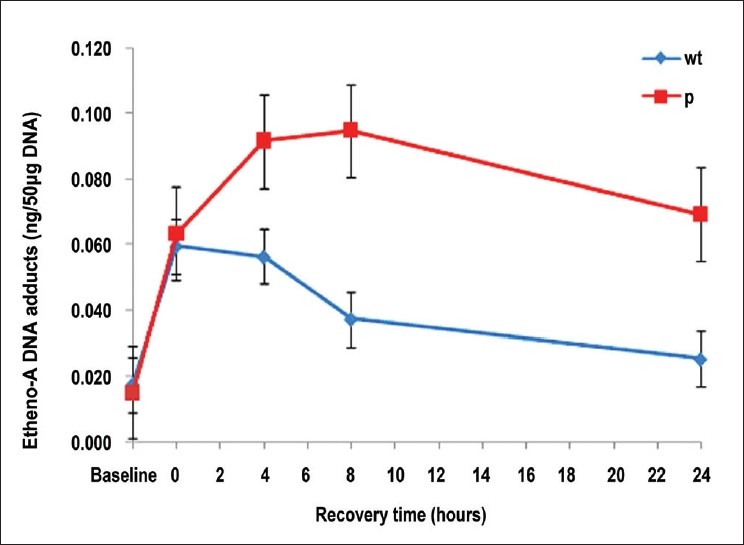
Plot of time course of average etheno-A DNA adduct levels for *XRCC1* homozygous wild-type cells (blue) and homozygous polymorphic variant cells (red) before and after exposure to the VC metabolite CEO

The results are presented in Table 1 and Figure 1. Mean adduct levels between the homozygous wild-type and homozygous variant groups were not statistically significantly different at the pre-exposure baseline, immediately post-exposure or after 4 h of recovery (*P* values 0.53, 0.85 and 0.14, respectively). However, after 8 and 24 h of recovery the polymorphic group had statistically significantly elevated adduct levels compared to the wild-type group (*P* values of 0.02 and 0.03, respectively). In fact, the adduct levels in the wild-type group had returned almost to the pre-exposure baseline levels at 24 h (approximately 82% repair efficiency on average). In comparison, the adduct levels in the polymorphic group were only at the immediate post-exposure levels after 24 h, which was approximately fourfold higher than the pre-exposure baseline levels, and by that time they had only declined marginally from the peak adduct levels recorded in that group at the 8 h post-recovery time point (approximately 32% repair efficiency on average). As can be seen in Figure 1, on average in the polymorphic group, adduct levels rose higher and declined more slowly than in the wild-type group, indicative of this less efficient repair of the induced etheno adducts.

**Table 1:** Etheno-A DNA Adduct Levels in XRCC1 399 Homozygous Wild-Type and Homozygous Polymorphic Variant Cells Following Treatment with the VC Metabolite CEO

XRCC1	Adduct Levels (ng/50 μg DNA)				
	
Genotype	Pre-Exposure	Post-Exposure Time Point (h)			
		
	Baseline	0	4	8	24
Wild-Type 1	0.021	0.073	0.065	0.033	0.027
Wild-Type 2	0.009	0.031	0.029	0.011	0.016
Wild-Type 3	0.020	0.082	0.069	0.046	0.029
Wild-Type 4	0.021	0.053	0.063	0.059	0.030
Wild-Type Average	0.018±0.006	0.060±0.012	0.056±0.019	0.037±0.021	0.025±0.006
Variant 1	0.017	0.089	0.128	0.124	0.097
Variant 2	0.016	0.067	0.091	0.079	0.070
Variant 3	0.013	0.035	0.057	0.082	0.041
Variant Average	0.015±0.002	0.063±0.027	0.092±0.025	0.095±0.025*	0.069±0.028*

* Variant versus Wild-type, P<0.05

## DISCUSSION AND CONCLUSIONS

These results suggest that the *XRCC1* codon 399 polymorphism can have a significant effect on the ability of cells to repair DNA damage caused by the active metabolites of VC, in particular the εA adducts known to be induced by CEO. This is consistent with other lines of evidence connecting this polymorphism with diminished DNA repair capability resulting in increased DNA mutation frequency and the occurrence of chromosomal aberrations and an increased risk of cancer.[11]

This is further supported by the fact that XRCC1 is known to participate in BER which is particularly efficient in the repair of εA adducts.[12] Although XRCC1 has no known enzymatic activity of its own, it is thought to function as a platform protein providing a scaffold that interacts with and coordinates the activity of the other proteins in the BER machinery, including glycosylases, an endonuclease (APE1), a DNA polymerase (Polβ), a ligase (LigIII) and poly(ADP-ribose)polymerases (PARP1 and PARP2).[8] Specific domains of XRCC1 are the sites of interaction with these other proteins. The XRCC1 central BRCT1 domain (approximately amino acid residues 315 - 403) has been associated with the functioning of PARP1, PARP2 and APE1.[13,14] Amino acid residue 399 lies within this central BRCT1 domain, and, hence, a polymorphism here might be expected to have some effect on the structure and function of the domain and its ability to interact with these proteins. Preliminary evidence from our laboratory on the molecular modeling of the normal and polymorphic XRCC1 proteins suggest that the 399 substitution does produce significant conformational changes in the BRCT1 domain, including the loss of secondary structural features such as α-helices that can be critical for protein-protein interactions.[15] Thus, these changes could disrupt XRCC1 interactions with PARP1, PARP2 and/or APE1 and the coordination of the BER machinery leading to the diminished repair capacity noted in cell culture. In fact, we had previously found similar results in cell culture of the *XRCC1* codon 399 polymorphism on the repair of εA adducts in lymphoblastoid cell lines exposed to CAA from a sibling pair, one of whom was homozygous wild-type and one of whom was homozygous variant;[16] in this case, the wild-type cells had a 91% repair efficiency compared to 22% efficiency in the variant cells (very similar to the average 82% and 32% repair efficiencies, respectively, found in this study).

These results also support the findings of our and other's studies of the effects of the *XRCC1* polymorphism on mutant biomarkers in VC workers and are consistent with the proposed carcinogenic pathway for VC.[6,7,17] As noted above, the reactive intermediates of VC, CEO and CAA, generate pro-mutagenic etheno-DNA adducts, including εA, that can cause the types of mutations (A->T transversions) seen in the *TP53* tumor suppressor gene found in the resultant ASLs, thus accounting for the presence of the mutant p53 biomarkers in VC-exposed workers. In these studies, the presence of the homozygous variant polymorphism at codon 399 increased the risk of occurrence of the mutant p53 biomarker by 1.9-to 3.9-fold, which is similar to the 2.6-fold difference in repair efficiency (82% vs. 32%) between the cell lines from these workers found in this study.[6,7,17] In summary, these results provide strong evidence for the biological plausibility of the epidemiologic observations, namely, that XRCC1 is involved in the repair of VC-induced lesions and that the polymorphism in *XRCC1* contributes significantly to an increased risk for mutagenic damage from VC and to the variability in susceptibility for such damage among exposed individuals. These findings have more general significance as well. For example, etheno-DNA adducts can be generated by exposures other than VC, XRCC1 participates in BER with many other glycosylases for repair of a range of different DNA damage, and *XRCC1* polymorphisms are quite common in many populations. Therefore, the overall contribution of *XRCC1* polymorphisms to susceptibility differences for mutagenesis and carcinogenesis could be substantial.

## DECLARATION OF COMPETING INTERESTS

The authors declare that they have no competing interests.

## AUTHOR'S PROFILE

**Paul W. Brandt-Rauf, Dr.P.H., M.D., Sc.D.,** is currently the Dean of the School of Public Health at the University of Illinois at Chicago. In addition, he holds appointments as Professor of Environmental and Occupational Health Sciences in the School of Public Health, Professor of Medicine in the College of Medicine, Professor of Bioengineering and Chemical Engineering in the College of Engineering, Professor of Earth and Environmental Sciences in the College of Liberal Arts and Sciences, and Professor of Public Administration in the College of Urban Planning and Public Affairs. He is also Professor Emeritus of Environmental Health Sciences, Medicine and Earth and Environmental Engineering at Columbia University where he was Chairman of the Department of Environmental Health Sciences until 2008. His major research interest is environmental carcinogenesis, particularly the molecular biology and molecular epidemiology of cancer-related proteins.
